# Activity of Omadacycline Alone and in Combination against Carbapenem-Nonsusceptible Acinetobacter baumannii with Varying Minocycline Susceptibility

**DOI:** 10.1128/spectrum.00542-22

**Published:** 2022-06-01

**Authors:** Taylor Abbey, Alesia Vialichka, Michele Jurkovic, Mark Biagi, Eric Wenzler

**Affiliations:** a College of Pharmacy, University of Illinois Chicago, Chicago, Illinois, USA; b College of Pharmacy, University of Illinois Chicago, Rockford, Illinois, USA; Univeristy of Texas Southwestern Medical Center

**Keywords:** susceptibility, time-kill assay, tetracycline, minocycline, omadacycline, synergy, combination therapy, pharmacodynamics, *Acinetobacter baumannii*, CRAB

## Abstract

Tetracycline-based combinations are increasingly used for serious carbapenem-nonsusceptible Acinetobacter baumannii (CNSAb) infections given their potent *in vitro* activity, synergism with other agents, and acceptable toxicity profile. Omadacycline is a novel aminomethylcycline with activity against minocycline-resistant pathogens, once daily oral dosing, and favorable pharmacokinetic properties. Given these potential advantages, the *in vitro* potency and antibacterial activity of omadacycline were evaluated alone and in combination against CNSAb with varying minocycline susceptibility. Broth microdilution testing of 41 CNSAb revealed that omadacycline (MIC_50/90_: 4/8 mg/L) inhibited 68.3% (28/41) of isolates at ≤4 mg/L and its activity was unaffected by minocycline nonsusceptibility (MIC_50/90_: 4/8 mg/L; 74.2% [23/31] inhibited at ≤4 mg/L). Ten (5 minocycline susceptible and 5 nonsusceptible) of the 41 CNSAb isolates were then evaluated in time-kill analyses against omadacycline and comparator agents alone and in dual- and triple-drug combinations at the free maximum concentration of drug in serum (*fC*_max_). Amikacin, meropenem, and polymyxin B alone were each bactericidal against 4 of 10 (40%) isolates while omadacycline and sulbactam were bactericidal against 0 (0%) and 1 (10%), respectively. In dual-drug combinations with omadacycline, synergy was observed against 80% of isolates with sulbactam followed by 30% with amikacin or polymyxin B and 0% with meropenem or rifampin. The triple-drug combination of omadacycline, sulbactam, and polymyxin B achieved synergy against just one additional strain over the omadacycline-sulbactam dual combination but significantly reduced the time to 99.9% kill by more than 6 h (4.6 ± 2.8 h vs. 11.3 ± 5.9 h, *P < *0.01). These results support the continued investigation into tetracycline-based combinations against CNSAb, particularly those including sulbactam, and suggest that omadacycline may have *in vitro* advantages over existing tetracycline-derivatives.

**IMPORTANCE** Treatment of infections due to Acinetobacter baumannii often involves the use of multiple antibiotics simultaneously as combination therapy, but it is unknown which antibiotics are best used together. Tetracycline agents such as minocycline and tigecycline maintain good activity against A. baumannii and are often used with one or more other agents to achieve better killing of the bacteria. Omadacycline is a new tetracycline that may have a role in the treatment of A. baumannii, but no data are available evaluating its interaction with other commonly used drugs such as polymyxin B and sulbactam. Therefore, the purpose of this study was to investigate the antibacterial activity of omadacycline when combined with one or more other agents against carbapenem-resistant strains of A. baumannii. These findings may then be used to design confirmatory studies that could help decide what drugs work best together and what combination of agents should be used for patients.

## INTRODUCTION

Carbapenem-nonsusceptible Acinetobacter baumannii (CNSAb) remains one of only two Gram-negative pathogens considered both an urgent threat nationally by the Centers for Disease Control and Prevention (CDC) and a critical priority internationally by the World Health Organization (1, 2). The exorbitant morbidity, mortality, and health care costs associated with CNSAb infections are due in large part to the insufficient number of available treatment options with adequate *in vitro* activity and appreciable clinical efficacy (3). In the United States, 18% of A. baumannii express the difficult-to-treat resistance phenotype and ≥40% are carbapenem nonsusceptible; only cefiderocol, the polymyxins, and the tetracycline-derivatives maintain reliable *in vitro* potency against this phenotype (4–7). Given the high rates of resistance, importance of time to effective therapy, and the lack of an established standard of care treatment regimen, combination therapy is routinely employed against A. baumannii and is supported by recommendations from the Infectious Diseases Society of America (8). Although clinical studies evaluating combination therapy are conflicting, preclinical data support combinations including a polymyxin with sulbactam, meropenem, rifampin, and/or a tetracycline derivative (9). Despite these data, the optimal combination of agents and dosing regimens to maximize efficacy and minimize toxicity have not been established. As attributable mortality rates for serious CNSAb infections are as high as 70% with current treatment and the prevalence and resistance continue to increase, it is crucial to continue to explore novel potential treatment regimens for this challenging pathogen (10–12).

Omadacycline is a novel aminomethylcycline with structural modifications at the C7 and C9 positions allowing it to circumvent the efflux pumps TetK and TetB and ribosomal protection protein mechanisms TetM and TetO that confer resistance to traditional tetracyclines including minocycline (13). These structural alterations also allow for once daily oral maintenance dosing making it only the second tetracycline-derivative after minocycline with activity against CNSAb available in oral formulation. Additional advantageous pharmacokinetic (PK) properties include significantly lower, concentration-independent protein binding and enhanced epithelial lining fluid penetration (14). Together these factors may make omadacycline a promising alternative to existing tetracycline derivatives for the treatment of A. baumannii, although supporting data are lacking. As such, the objective of this study was to evaluate the *in vitro* potency of omadacycline and comparator agents against A. baumannii via broth microdilution (BMD) testing and assess the antibacterial activity of each agent alone and in two- and three-drug combinations in time-kill analyses.

## RESULTS

### Susceptibility testing.

**FIG 1:**
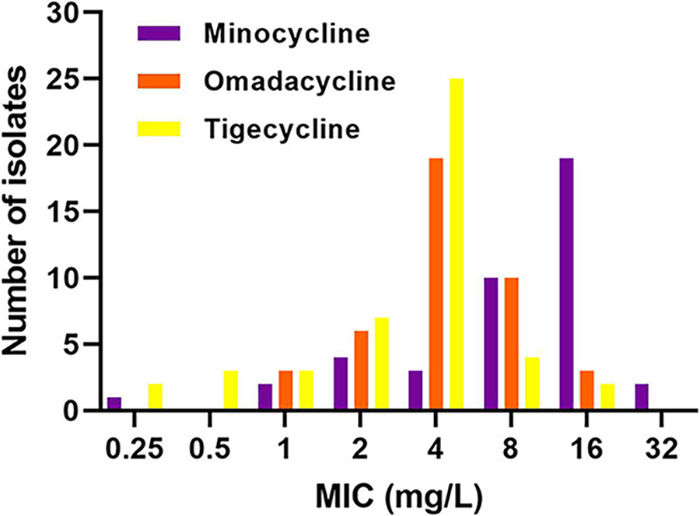
MIC distributions of minocycline, omadacycline, and tigecycline against 41 clinical carbapenem-nonsusceptible Acinetobacter baumannii.

Genotypically, 97.6% (40/41) of CNSAb isolates tested harbored at least 1 aminoglycoside-modifying enzyme and at least 1 Ambler class D *bla*OXA gene (most commonly *bla*OXA-23 at 48.8%), while all 41 (100%) carried the class C gene *bla*ADC-25. Nineteen of 41 (46%) also coharbored a class A *bla*TEM-1B or -1D gene while 32/41 (78%) and 1/41 (2.4%) carried the *tet*(B) and *tet*(A) efflux genes, respectively. The phenotypic MIC_50_, MIC_90_, MIC range, and percent susceptible for each agent, as applicable, against all 41 CNSAb isolates are summarized in Table 1. Only 12.2% (5/41) of isolates were susceptible to amikacin and none (0%) were susceptible to meropenem (1 intermediate [2.4%] and 40 [97.6%] resistant). Similarly, activity of sulbactam was poor with MIC_50/90_ values of 32/128 mg/L and just 1 isolate testing susceptible. Polymyxin B displayed the lowest MIC_50/90_ values of any agent at 0.5/0.5 mg/L although no isolates were considered susceptible as the revised CLSI interpretive criteria only include intermediate and resistant breakpoints for the polymyxins. Minocycline displayed the highest rate of susceptibility overall at 29.3% (12/41) although MIC_50/90_ values were 16/16 mg/L compared to omadacycline and tigecycline each at 4/8 mg/L. Against the 31 minocycline nonsusceptible isolates, omadacycline and tigecycline MIC_50/90_ were unchanged at 4/8 mg/L each and 74.2% and 90.3% were inhibited at ≤4 mg/L of omadacycline and tigecycline, respectively. The MIC distributions for each tetracycline derivative against all 41 CNSAb isolates are overlaid in Fig. 1. Overall, isolate AB4 was the most resistant and displayed the highest MICs across all eight agents tested followed by AB6. Isolate AB7 and AB8 were the least resistant of the 10 tested although each were susceptible only to minocycline.

**TABLE 1:** Activity of omadacycline and comparator agents against clinical carbapenem-nonsusceptible Acinetobacter baumannii isolates (*n* = 41)^*a*^

	MIC (mg/L)			Susceptibility (%)		
Agent	50%	90%	Range	S	I	R
Amikacin	128	>256	2 to >256	12.2	24.4	63.4
Meropenem	≥256	≥256	4 to ≥256	0	2.4	97.6
Minocycline	16	16	0.25 to 32	29.3	19.5	51.2
Omadacycline	4	8	1 to 16	NC	NC	NC
Polymyxin B	0.5	0.5	0.125 to ≥128	NC	95.1	4.9
Rifampin	16	64	4 to >256	NC	NC	NC
Sulbactam	32	128	2 to 128	2.4	0	97.6
Tigecycline	4	8	1 to 16	NC	NC	NC

a S, susceptible; I, intermediate; R, resistant; NC, no applicable interpretive criteria available.

### Time-kill experiments.

Table 2 displays the MIC values for each agent against the 10 CNSAb selected for time-kill experiments. Comparing the MIC values in Table 2 to the simulated free maximum concentrations of drug in serum (*fC*_max_) as shown in Table 3 reveals that, although only 3/10 (30%) isolates were considered susceptible to amikacin, 7/10 (70%) had an MIC at or below the *fC*_max_ of 55.5 mg/L. All 10 (100%) isolates were considered nonsusceptible to meropenem although 3/10 (30%) had MICs less than or equal to *fC*_max_ (40 mg/L) compared to sulbactam for which all (100%) were considered resistant while 7/10 (70%) had an MIC less than or equal to *fC*_max_ (60.8 mg/L). The vast majority (80%) of polymyxin MICs were 5 to 10-fold below the *fC*_max_ (2.61 mg/L) though none were considered susceptible. Finally, just 1/10 (10%) minocycline isolates had an MIC approximately equal to its *fC*_max_ (0.24 mg/L) versus 2/10 (20%) for omadacycline (1.21 mg/L) and 0/10 (0%) for tigecycline (0.08 mg/L).

**TABLE 2:** MICs and susceptibility interpretation of omadacycline and comparator agents against 10 carbapenem-nonsusceptible clinical A. baumannii isolates included in time-kill experiments^*a*^

Isolate	Amikacin		Meropenem		Minocycline		Omadacycline		Polymyxin B		Rifampin		Sulbactam		Tigecycline	
AB1	32	I	16	R	0.25	S	4	NC	0.5	I	4	NC	128	R	8	NC
AB2	32	I	≥256	R	2	S	16	NC	0.25	I	16	NC	32	R	8	NC
AB3	8	S	4	I	16	R	8	NC	0.5	I	16	NC	32	R	4	NC
AB4	>256	R	128	R	16	R	16	NC	>64	R	>256	NC	16	R	16	NC
AB5	32	I	≥256	R	16	R	1	NC	0.25	I	16	NC	64	R	2	NC
AB6	>256	R	≥256	R	8	I	4	NC	>64	R	16	NC	16	R	4	NC
AB7	32	I	≥256	R	1	S	2	NC	0.25	I	8	NC	16	R	2	NC
AB8	>256	R	≥256	R	4	S	4	NC	0.25	I	4	NC	32	R	2	NC
AB9	2	S	16	R	8	I	1	NC	0.25	I	8	NC	16	R	1	NC
AB10	4	S	64	R	2	S	8	NC	0.5	I	8	NC	64	R	4	NC

a Gray shaded cells represents isolates for which the respective drug’s free maximum concentration of drug in serum (*fC*_max_) was ≥MIC. NC, no applicable interpretive criteria.

**TABLE 3:** Representative doses and *fC*_max_ values simulated for each agent in time-kill experiments

Agent	Dose^*a*^	*C*_max_ (mg/L)	Protein binding (%)	*fC*_max_ (mg/L)	Reference
Amikacin	15 mg/kg i.v. over 1 h	60	7.5	55.5	(54)
Meropenem	2 g i.v. over 3 h	40.9	2	40	(55)
Minocycline	100 mg i.v. over 1 h	0.99	76	0.24	(56, 57)
Omadacycline	100 mg i.v. over 30 min	1.51	21	1.21	(14)
Polymyxin B	1.5 mg/kg i.v. over 1 h	6.21	58	2.61	(58)
Rifampin	300 mg i.v. over 30 min	8.90	80	1.78	(59)
Sulbactam	1 g i.v. over 30 min	98	38	60.8	(60)
Tigecycline	50 mg i.v. over 30 min	0.38	80	0.08	(61)

a Single doses.

**FIG 2:**
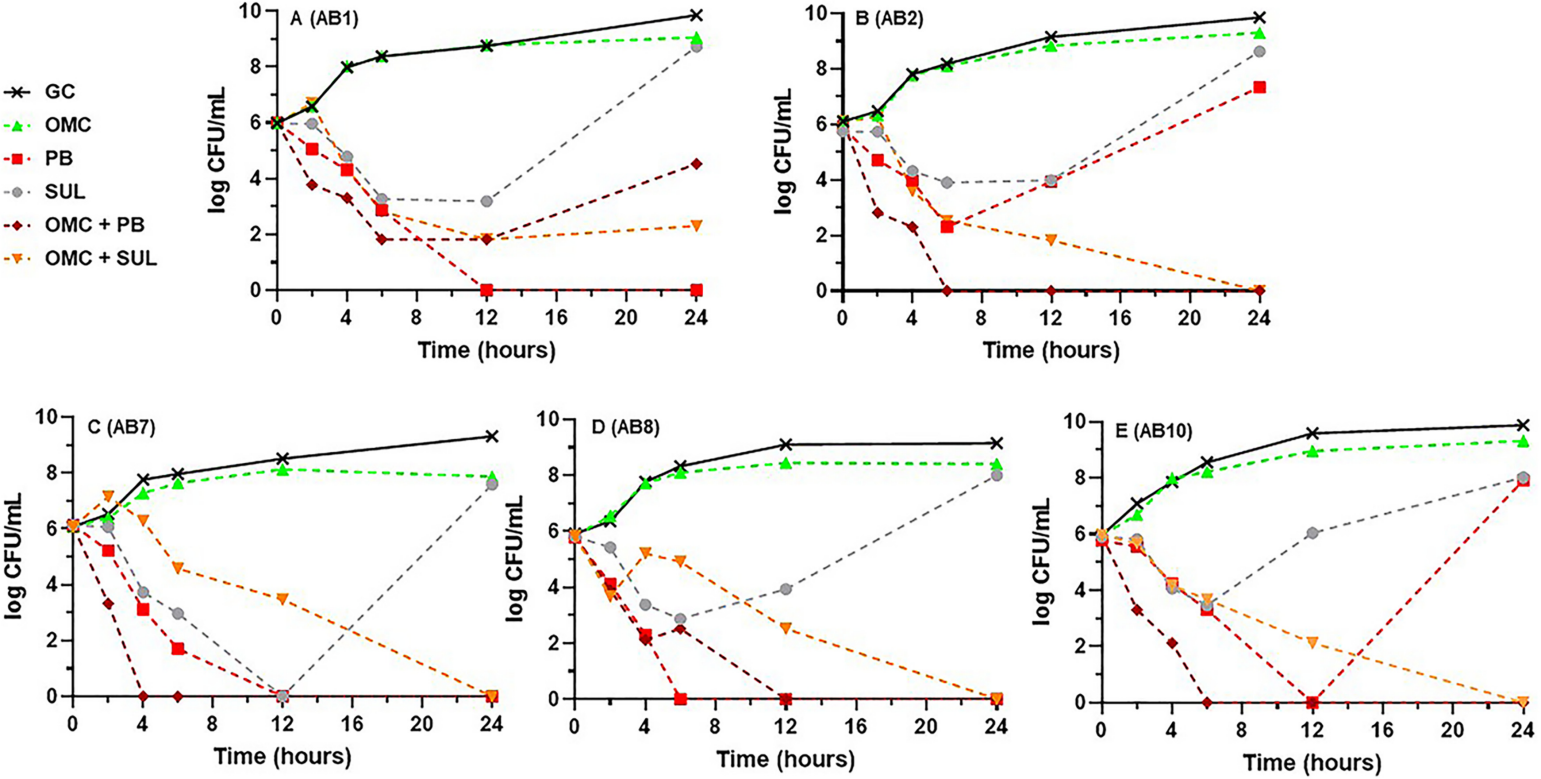
Mean log_10_ CFU/mL versus time profiles for omadacycline, polymyxin B, and sulbactam alone versus each respective omadacycline-based dual drug combination at the free maximum concentration of drug in serum (*fC*_max_) against each of the 5 minocycline susceptible A. baumannii strains. Curves represent average concentrations from triplicate experiments. (A) AB1. (B) AB2. (C) AB7. (D) AB8. (E) AB10. GC, growth control; OMC, omadacycline; PB, polymyxin B; SUL, sulbactam.

**FIG 3:**
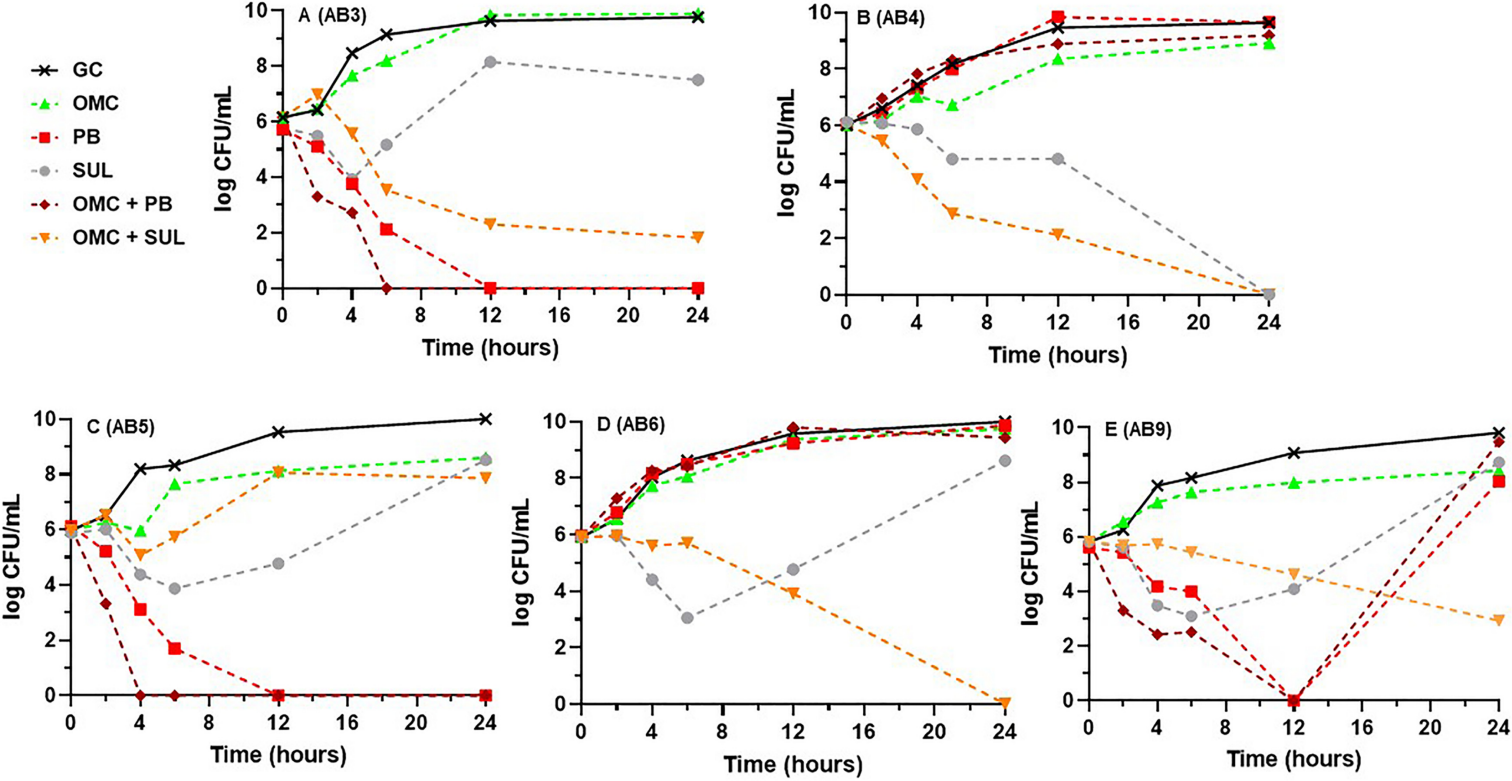
Mean log_10_ CFU/mL versus time profiles for omadacycline, polymyxin B, and sulbactam alone versus each respective omadacycline-based dual drug combination at *fC*_max_ against each of the 5 minocycline-nonsusceptible A. baumannii strains. Curves represent average concentrations from triplicate experiments. (A) AB3. (B) AB4. (C) AB5. (D) AB6. (E) AB9.

Results of time-kill experiments for omadacycline, polymyxin B, and sulbactam alone and in their respective dual omadacycline-based combinations at *fC*_max_ are displayed in Fig. 2 against the five minocycline susceptible isolates and in Fig. 3 against the nonsusceptible isolates. Figures S1 and S2 in the supplemental material display the single- and dual-drug combination results for the other agents tested against the minocycline susceptible and nonsusceptible A. baumannii strains, respectively. Alone, amikacin, meropenem, and polymyxin B were each bactericidal against 4 of 10 (40%) strains. Sulbactam was bactericidal against one strain (10%) while the tetracycline derivatives and rifampin were not bactericidal against any (0%) and 24-h bacterial densities were similar to the drug-free control. Omadacycline with sulbactam was the most active dual combination resulting in synergy against 8/10 (80%) strains (all 5 minocycline susceptible and 3 of 5 nonsusceptible), improvement in bactericidal activity from just 1/10 (10%) with sulbactam alone to 8/10 (80%), and achievement of eradication against 5/10 (50%) tested strains. It was also the only dual combination to have any activity or achieve bactericidal activity against the challenging AB4 and AB6 strains (Fig. 3B and D). The mean (±SD) log_10_ CFU/mL decrease after exposure to the omadacycline plus sulbactam combination from 0 to 24 h across all 10 isolates was 4.24 ± 2.51. Omadacycline in combination with either amikacin or polymyxin B was synergistic against 3/10 (30%) strains each (2 minocycline susceptible and 1 nonsusceptible), bactericidal against 6/10 (60%) each, and achieved eradication against 5/10 (50%) and 6/10 (60%), respectively. The only instance of antagonism occurred with the combination of omadacycline and polymyxin B compared to polymyxin B alone against AB1 (Fig. 2A; mean 24-h log_10_ CFU/mL increase from 0 to 4.53). Omadacycline plus rifampin was synergistic and bactericidal against only 2/10 (20%) strains and omadacycline plus meropenem was synergistic against just 1/10 (10%) isolates and did not improve bactericidal activity over meropenem alone (40%) (Fig. S1 and S2). Lastly, the dual combination of meropenem plus polymyxin B was also evaluated against the four strains for which neither meropenem or polymyxin B alone was bactericidal (AB2, 4, 5, and 6). This combination achieved synergy and bactericidal activity against all four strains with a mean (±SD) log_10_ CFU/mL decrease from 0 to 24 h of 4.77 ± 2.48.

**FIG 4:**
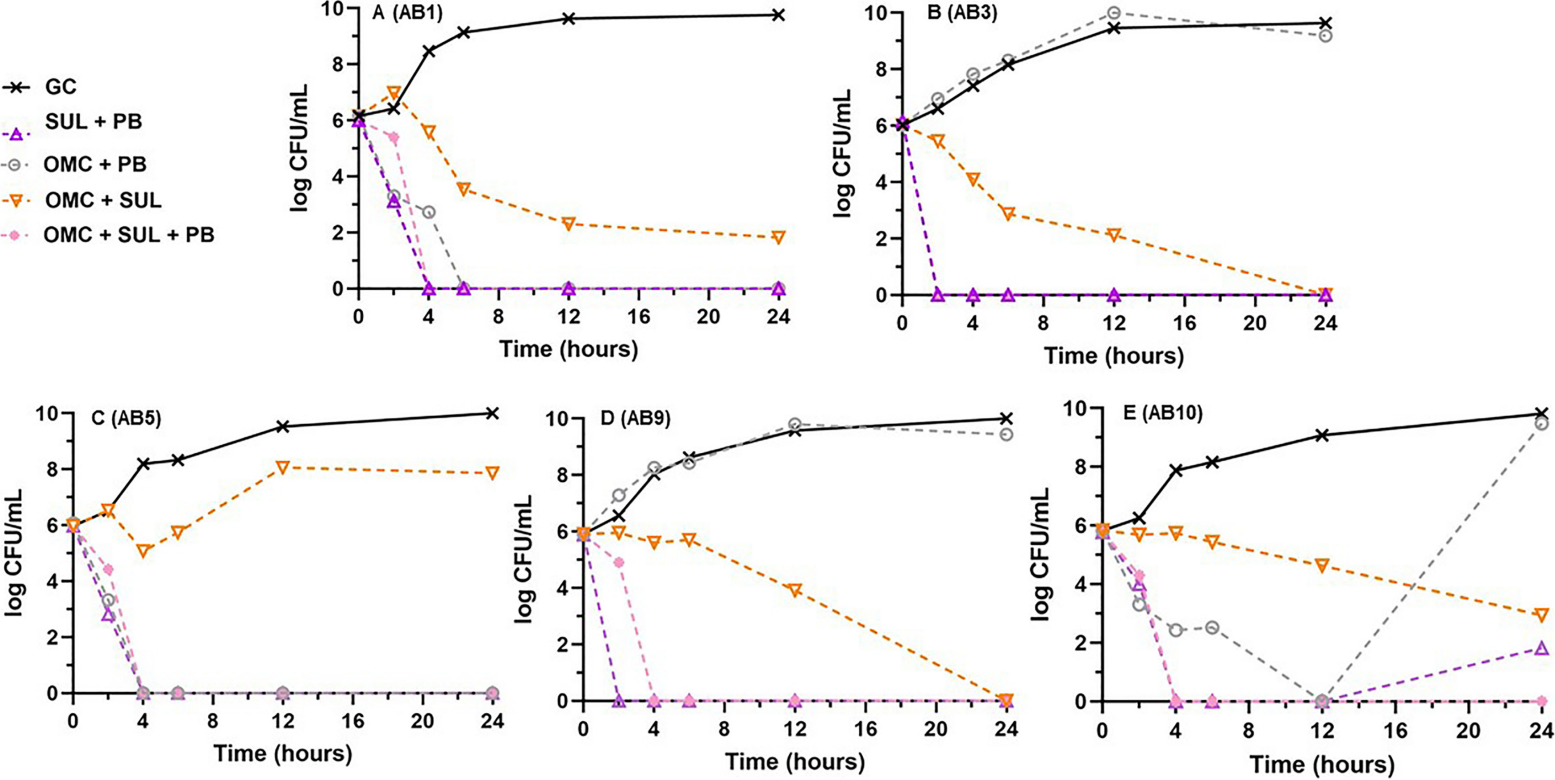
Mean log_10_ CFU/mL versus time profiles for the triple combination of omadacycline plus sulbactam plus polymyxin B versus each respective two drug combination against a representative subset of 5 A. baumannii strains (2 minocycline susceptible, 3 nonsusceptible). Curves represent average concentrations from triplicate experiments. (A) AB1. (B) AB3. (C) AB5. (D) AB9. (E) AB10. AMK, amikacin; MER, meropenem; RIF, rifampin.

Based on results from the two-drug combination experiments, the triple combination of omadacycline plus sulbactam plus polymyxin B was evaluated to assess whether the antibacterial activity could be improved over the omadacycline plus sulbactam two drug combination. Consequently, the dual combination of sulbactam plus polymyxin B was tested against all 10 strains to allow for comparison to the triple combination and demonstrated 50% synergy (3 minocycline susceptible and 2 nonsusceptible) and 100% bactericidal activity (10/10), which was improved over 4/10 (40%) for polymyxin B alone. The mean (±SD) log_10_ CFU/mL decrease after exposure to the sulbactam plus polymyxin B combination from 0 to 24 h across all 10 isolates was 5.8 ± 0.64. Figure 4 displays the results of the omadacycline plus sulbactam plus polymyxin B triple combination versus the three comparative dual combinations against a representative subset of five strains (minocycline susceptible strains AB1 and AB10 and nonsusceptible strains AB3, AB5, and AB9). The triple combination of omadacycline plus sulbactam plus polymyxin B achieved synergy against only one (10%) additional strain (AB5; 9/10) over the omadacycline plus sulbactam combination (8/10). The triple combination did achieve a ≥3 log_10_ CFU/mL reduction almost 7 h sooner on average than the omadacycline and sulbactam dual combination (4.6 ± 2.8 h versus 11.3 ± 5.9 h, *P < *0.01). Additionally, eradication was increased from 5/10 (50%) with omadacycline plus sulbactam to 10/10 (100%) and was not affected by susceptibility to minocycline. The mean (±SD) log_10_ CFU/mL decrease after exposure to the omadacycline plus sulbactam plus polymyxin B triple combination from 0 to 24 h across all 10 isolates was 5.99 ± 0.11. While the activity of the triple combination was improved over that of either the omadacycline plus sulbactam or omadacycline plus polymyxin B dual combinations, it was virtually indistinguishable from the sulbactam plus polymyxin B dual combination (Fig. 4).

## DISCUSSION

The inability to achieve bactericidal activity *in vitro*, the obstinate link between resistance and delays in time to effective antimicrobial therapy, and the high mortality rates associated with monotherapy against CNSAb strongly support the use of combination therapy (8, 15). Even when faced with favorable MICs, monotherapy is often inadequate as was demonstrated well in the recent cefiderocol CREDIBLE-CR study in which 85% of patients received cefiderocol monotherapy versus 72% combination therapy in the best available therapy (BAT) group (16). Despite 92% of A. baumannii cefiderocol MICs being ≤2 mg/L, 28-day mortality was twice as high for cefiderocol compared to the BAT group. Although combination therapy is standard and endorsed by national and international societies and guidelines, the optimal combination for serious CNSAb infections has remained elusive (3, 8). As the traditional polymyxin-carbapenem combination has fallen out of favor due to refuting controlled trial data and concerns over pharmacokinetic-pharmacodynamic (PK/PD), toxicity, and the development of carbapenem resistance, investigation into alternative combination regimens for CNSAb is sorely needed (17–26).

The novel aminomethylcycline omadacycline is a welcomed addition to the available tetracycline derivatives given its potency against TetB positive, minocycline-resistant CNSAb (~70% of A. baumannii), once daily oral dosing, and favorable PK properties including low protein binding and enhanced intrapulmonary penetration (14, 27, 28). Tetracycline derivatives, including omadacycline, have demonstrated little antibacterial activity against A. baumannii alone in *in vitro* pharmacodynamic models, as expected given their low free serum concentrations and bacteriostatic nature (29–31). Conversely, tetracycline-based combinations often produce the most potent synergy and bactericidal activity *in vitro* and *in vivo* versus nontetracycline-based combinations and available comparative clinical data are encouraging (8, 32–36). In the present study, omadacycline was evaluated alone and in combination for the first time in six different two- and three-drug combinations against CNSAb with varying minocycline susceptibility. Broth microdilution testing of omadacycline demonstrated the second lowest MIC_50/90_ values at 4/8 mg/L behind polymyxin B despite intentionally enriching our sample with minocycline nonsusceptible isolates (Table 2 and Fig. 1). The *in vitro* potency observed herein is consistent with previous analyses of omadacycline against larger samples of A. baumannii (37–39). Bactericidal activity was achieved against ≤4 of 10 isolates for any agent in alone in time-kill analyses, in line with the high rates of nonsusceptibility observed among the selected isolates (Table 3). Omadacycline-based combinations were tested in 50 separate time-kill experiments overall (5 per isolate), demonstrating synergy with another agent in 17/50 (34%) and bactericidal activity in 26/50 (52%). Across the 10 isolates included, the highest rate of synergy and degree of bactericidal activity was observed when omadacycline was combined with sulbactam (80%), followed by omadacycline with amikacin or polymyxin B (30% each). The addition of polymyxin to the omadacycline-sulbactam dual combination resulted in more rapid bactericidal activity, although this mimicked the activity of the dual polymyxin-sulbactam combination which was less synergistic (50% versus 80%) than omadacycline-sulbactam. While previous *in vitro*, *in vivo*, and clinical data have demonstrated compelling synergy between tetracyclines and sulbactam, this combination is almost never employed clinically despite the added benefit of reduced toxicity compared to polymyxin-based regimens (20, 30, 40–44). Our work adds to the existing literature supporting the further exploration of tetracycline-sulbactam combinations and adds the first set of data evaluating omadacycline in combination against A. baumannii.

Strengths of our study include the use of a broad panel of clinical A. baumannii isolates with varying minocycline susceptibilities and the evaluation of omadacycline alone and in combination with currently preferred agents. Limitations are primarily related to the inherently static, *in vitro* nature of time-kill experiments including the inability to simulate human PK parameters and other physiologic factors such as protein binding. Nonetheless, time-kill assays are well recognized as suitable and efficient tools for assessing antibacterial activity and PD drug interactions of antibiotic combinations (45). Additionally, as the current study was focused on omadacycline-based combinations, not all possible dual and/or triple combinations were evaluated including some that have demonstrated synergy in previous studies (46). Recent findings suggest rifabutin may be significantly more potent than rifampin against A. baumannii, although this was only evident when testing in nutrient-depleted, mammalian cell culture media, which would not be suitable for the other agents included in this study (47). Finally, testing agents at *fC*_max_ could have overestimated the antibacterial activity and limited comparability between agents versus using MIC multiplicatives although MICs were greater than *fC*_max_ in the majority of experiments and bactericidal activity was rare in single-drug time-kill experiments.

In conclusion, omadacycline displays potent *in vitro* activity against CNSAb including strains that harbor TetB and are minocycline resistant. Omadacycline in combination with sulbactam was synergistic and bactericidal against 8/10 (80%) isolates, including strains that were nonsusceptible to every drug tested. In agreement with the growing body of data supporting tetracycline-based combinations against CNSAb, this work adds further impetus to continue to explore tetracycline-sulbactam combinations as a promising regimen toward the goal of maximize antimicrobial efficacy and minimizing toxicity against this challenging pathogen.

## MATERIALS AND METHODS

### Bacteria and susceptibility testing.

A total of 41 genotypically characterized clinical A. baumannii isolates were selected from the CDC & FDA Antibiotic Resistance Isolate Bank to encompass a range of phenotypes against the agents tested, particularly the tetracycline derivatives (48). Complete genomes were downloaded from the NCBI nucleotide database and resistance genes were identified by BLAST searching against ResFinder 3.1 and CARD-RGI databases (49, 50). Isolates were maintained at −80°C in cation-adjusted Mueller-Hinton broth (CAMHB; Teknova, Hollister, CA, USA) with 20% glycerol and were subcultured twice on tryptic soy agar plates with 5% sheep blood prior to use.

Analytical grade amikacin, meropenem, minocycline, polymyxin B, rifampin, sulbactam, and tigecycline powders were obtained commercially (Sigma-Aldrich, St. Louis, MO, USA). Sulbactam was tested alone as ampicillin has no activity against A. baumannii nor does it impact the activity of sulbactam (51). Analytical grade omadacycline powder was provided by the manufacturer (Paratek Pharmaceuticals, Boston, MA, USA). Stock solutions of each agent were freshly prepared as single-use aliquots at the beginning of each week and kept frozen at −80°C. CAMHB was freshly prepared and used within 12 h of preparation. MICs were determined in triplicate via reference BMD according to Clinical and Laboratory Standards Institute (CLSI) guidelines using the same 0.5 McFarland suspension on the same day (52). Modal MIC values from triplicate BMDs were recorded and are reported as MIC_50_, MIC_90_, and MIC range. Escherichia coli ATCC 25922 and Pseudomonas aeruginosa ATCC 27853 were used as quality control organisms. Susceptibility interpretations were based on CLSI criteria against A. baumannii where available (52).

### Time-kill experiments.

Time-kill experiments were performed in triplicate on the same day against a subset of 10 CNSAb isolates purposefully selected from the original group of 41. These 10 isolates were chosen based on their minocycline susceptibility (5 susceptible and 5 nonsusceptible) and to ensure that each log_2_ omadacycline BMD MIC across the observed range (1–16 mg/L) was represented at least once. Experiments were performed according to CLSI guidelines modified using a final volume of 2 mL in deep-well, nontissue-treated plates (20). A starting inoculum of ~10^6^ CFU/mL was prepared by suspending three to four isolated colonies selected from a pure overnight culture in 5 mL of sterile saline and adjusting to 0.5 McFarland standard, which was subsequently incubated with agitation to ensure log-phase growth and then diluted 1:100 in CAMHB. Colony counts were performed to ensure final inoculum densities. Time-kill experiments were performed with each agent at its representative plasma *fC*_max_ concentration after standard dosing as displayed in Table 3. Single-drug experiments were performed for each agent followed by dual combinations of omadacycline with amikacin, meropenem, polymyxin B, rifampin, and sulbactam. Additionally, triple combinations of omadacycline plus polymyxin B plus either meropenem, rifampin, or sulbactam were tested based on results from the dual combination experiments. A growth control without any antibiotic was included with each experiment. At the prespecified time points of 0, 2, 4, 6, 12, and 24 h, aliquots of 20 μL were removed from the suspensions and serially diluted in log_10_ dilutions. A 50-μL aliquot was then plated on MH agar plates using an automated spiral plater (Don Whitley WASP Touch, Microbiology International, Frederick, MD) and incubated at 35°C for at least 24 h prior to enumeration. Colony counts were performed using an automated colony counter (ProtoCOL 3 Plus, Synbiosis, Frederick, MD). The theoretical lower limit of quantitation was 100 CFU/mL. Time-kill curves were generated by plotting the average log_10_ CFU/mL versus time to compare the 24-h killing effects of drugs alone and in dual and triple combinations. Bactericidal activity was defined as ≥3 log_10_ CFU/mL reduction at 24 h compared to the starting inoculum. Synergy was defined as ≥2 log_10_ CFU/mL reduction at 24 h compared to the most active drug alone for dual combination experiments and versus the most active dual combination for triple combination experiments. Antagonism was defined as ≥2 log_10_ CFU/mL increase at 24 h compared to the most active drug alone or dual combination (53).
