# Supplementary material for: Activity of Omadacycline Alone and in Combination against Carbapenem-Nonsusceptible Acinetobacter baumannii with Varying Minocycline Susceptibility
Source: Microbiol Spectr. 2022 Jun 1;10(3):e00542-22. doi: 10.1128/spectrum.00542-22 (PMC9241703; doi:10.1128/spectrum.00542-22)

Figure S1. Mean $\log_{10}$ CFU/mL vs. time profiles for omadacycline, amikacin, meropenem, and rifampin alone vs. each respective omadacycline-based dual drug combination at *f*Cmax against each of the 5 minocycline susceptible *A. baumannii* strains. Curves represent average concentrations from triplicate experiments. Abbreviations: GC, growth control; OMC, omadacycline; AMK, amikacin; MER, meropenem; RIF, rifampin.

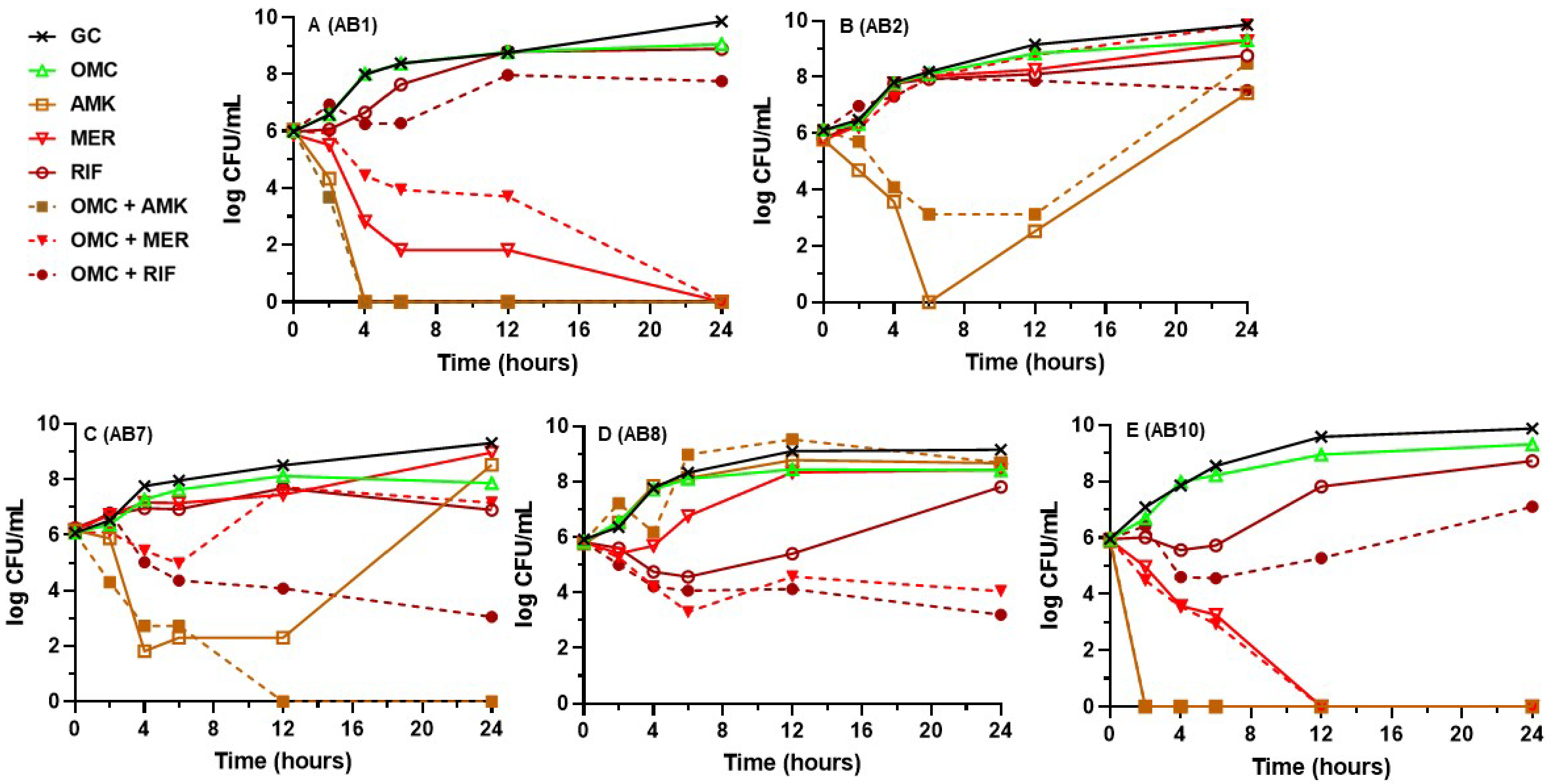

Figure S2. Mean $\log_{10}$ CFU/mL vs. time profiles for omadacycline, amikacin, meropenem, and rifampin alone vs. each respective omadacycline-based dual drug combination at *f*Cmax against each of the 5 minocycline-non-susceptible *A. baumannii* strains. Curves represent average concentrations from triplicate experiments. Abbreviations: GC, growth control; OMC, omadacycline; AMK, amikacin; MER, meropenem; RIF, rifampin.

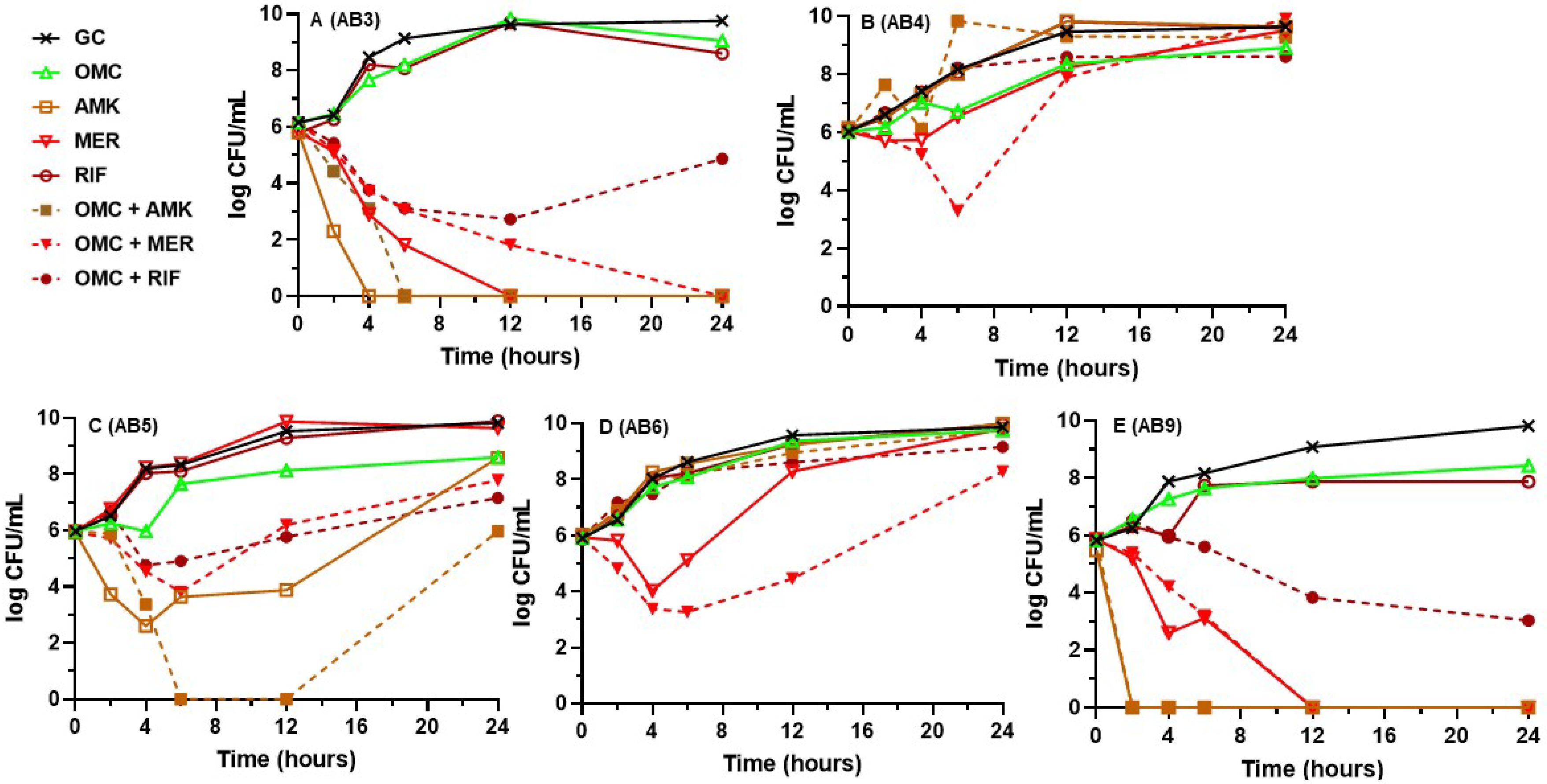

Supplement: SUPPLEMENTAL FILE 1 — Supplemental material. Download spectrum.00542-22-s0001.pdf, PDF file, 0.8 MB [file spectrum.00542-22-s0001.pdf]
